# Effect of timing of coronary revascularization in patients with post-infectious myocardial infarction

**DOI:** 10.1371/journal.pone.0272258

**Published:** 2022-08-18

**Authors:** Chuan-Tsai Tsai, Ya-Wen Lu, Ruey-Hsing Chou, Chin-Sung Kuo, Po-Hsun Huang, Cheng-Hsueh Wu, Shao-Sung Huang

**Affiliations:** 1 Division of Cardiology, Department of Medicine, Taipei Veterans General Hospital, Taipei, Taiwan; 2 Cardiovascular Research Center, National Yang-Ming Chiao Tung University, Taipei, Taiwan; 3 Department of Critical Care Medicine, Taipei Veterans General Hospital, Taipei, Taiwan; 4 Institute of Clinical Medicine, National Yang-Ming Chiao Tung University, Taipei, Taiwan; 5 Division of Endocrinology and Metabolism, Department of Medicine, Taipei Veterans General Hospital, Taipei, Taiwan; Public Library of Science, UNITED KINGDOM

## Abstract

**Objectives:**

Acute infection is a well-known provocative factor of acute myocardial infarction (AMI). Prognosis is worse when it is associated with sepsis. Coronary revascularization is reported to provide benefit in these patients; however, the optimal timing remains uncertain.

**Methods:**

This retrospective study was performed at a tertiary center in Taipei from January 2010 to December 2017. 1931 patients received coronary revascularization indicated for AMI. Among these, 239 patients were hospitalized for acute infection but later developed AMI. Patients with either an ST-elevation myocardial infarct or the absence of obstructive coronary artery disease were excluded. Revascularization was performed via either percutaneous coronary intervention (PCI) or coronary artery bypass graft (CABG). We defined early and delayed revascularization groups if it was performed within or after 24 hours of the diagnosis of AMI, respectively. We evaluated whether the timing of revascularization altered 30-day and one-year all-cause mortality.

**Results:**

At one month, 24 (26%) patients died in early revascularization group and 32 (22%) patients in delayed revascularization group. At one year, 40 (43%) and 59 (40%) patients died on early and delayed revascularization groups respectively. Early revascularization did not result in lower 30-day all-cause mortality (P = 0.424), and one-year all-cause mortality (Hazard ratio (HR): 0.935; 95% confidence interval (CI): 0.626–1.397, P = 0.742) than delay revascularization.

**Conclusions:**

Timing of coronary revascularization of post infectious acute coronary syndrome may be arranged according to individual risk category as those without sepsis.

## Introduction

Acute myocardial infarction (AMI) is the leading cause of death worldwide [1]. Pathogenesis includes atherosclerotic plaque rupture, ulceration, dissection resulting in intraluminal thrombosis [2]. Acute infection, especially in the upper respiratory tract, increases the short-term risk of AMI, particularly during the first three days of illness [3,4]. The mechanisms underlying post-infectious AMI include coronary endothelial dysfunction [5], platelet activation leading to coronary artery thrombosis [6]. However, sepsis-related increased myocardial oxygen consumption can cause a type 2 MI. Type 2 myocardial infarction is an emerging pathophysiological condition due to a mismatch between myocardial oxygen supply and demand, leading to ischemic injury [7]. Additionally, in-hospital mortality is higher in patients with AMI, complicated by sepsis, than those without sepsis [8].

Invasive revascularization procedure such as percutaneous coronary intervention (PCI) or coronary artery bypass graft (CABG) improved outcome compared with conservative management in post-infectious AMI [9]. Emergent coronary revascularization within 60 minutes of first medical contact is the preferred reperfusion strategy for ST-elevation myocardial infarction. Immediate (< 2 hours), early (< 24 hours), or selective invasive reperfusion (≥ 24 hours) are treatment options for non-ST elevation myocardial infarction, depending upon the patients’ risk category [10]. However, optimal timing of invasive management for post-infectious AMI is not well investigated. We performed this retrospective study to evaluate the effect of early (within 24 hours of the diagnosis of AMI) and delayed (after 24 hours) invasive management on short-term and long-term all-cause mortality rates in patients with AMI and laboratory-documented sepsis.

## Material and methods

**Fig 1:**
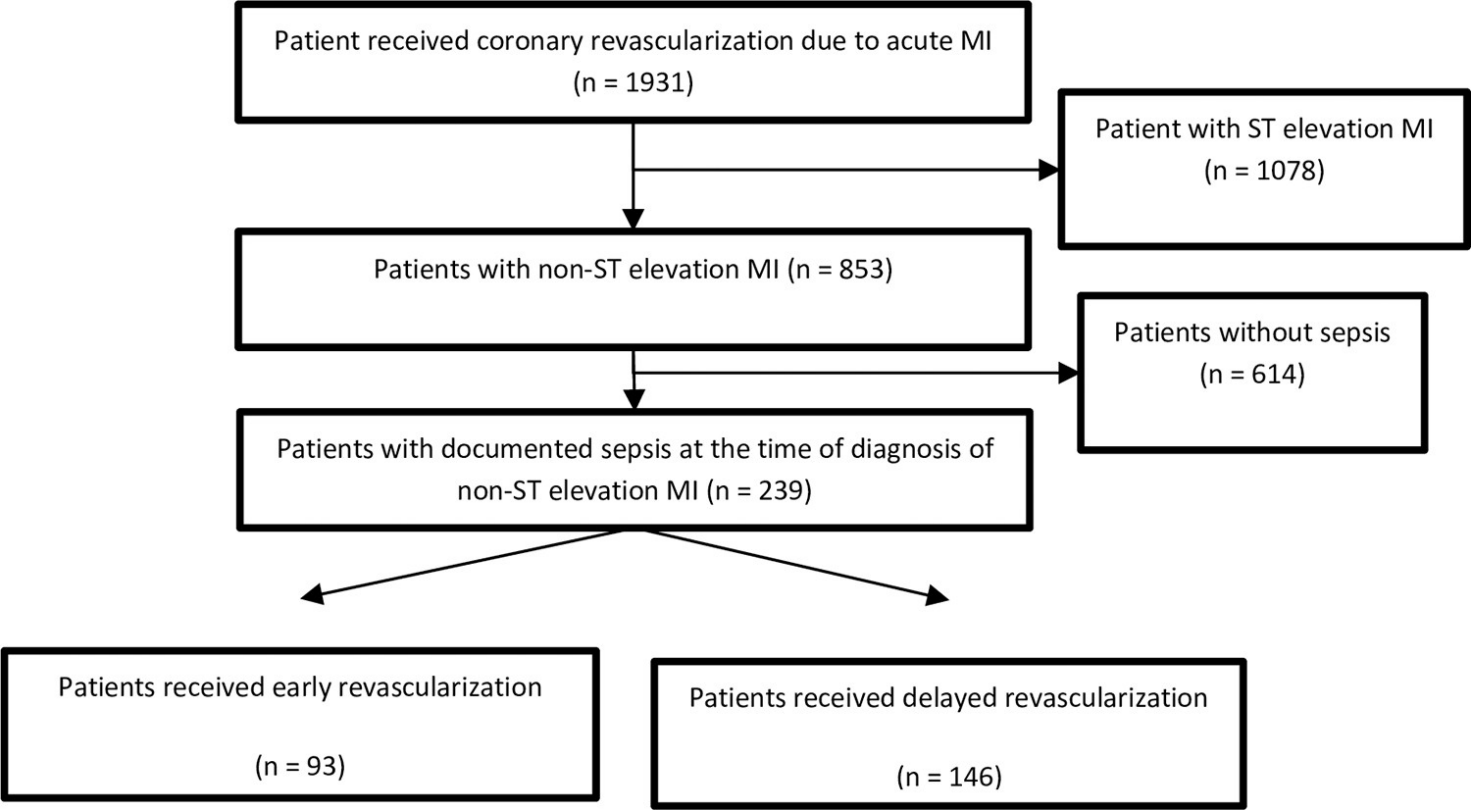
Flowchart of patient selection.

This is a retrospective study which was conducted in Taipei Veterans General Hospital, a tertiary medical center in Taiwan. From January 2010 to December 2017, we identified 1931 patients, ≥ 18 years of age, who received coronary revascularization indicated for AMI. Of these, 1081 patients were excluded for ST-elevation myocardial infarction. We reviewed electronic medical records of 853 patients with non-ST elevation myocardial infarction. A total of 239 patients with laboratory-confirmed sepsis who later developed AMI were included in our study. A flowchart of patient enrollment is shown in Fig 1. This study was approved by our hospital institutional review board and compliant with the Declaration of Helsinki.

### Definitions

AMI was defined by detection of the rise or fall of cardiac biomarkers (troponins) above the upper reference limit with at least one of the followings; symptoms of ischemia, new significant ST-segment changes or left bundle branch block; development of pathological Q waves; imaging evidence of either a new loss of viable myocardium or a new regional wall motion abnormality [2]. Post-infectious AMI was defined as an AMI and the concomitant diagnosis of acute infection at the onset of AMI symptoms. Acute infection was defined as the diagnosed or suspected focal infection with some of the followings: abnormal general variables (fever, hypothermia, tachypnea); inflammatory markers (leukocytosis, elevated C-reactive protein, and/or procalcitonin); hemodynamic variables (hypotension; organ dysfunction [e.g., hypoxemia, acute oliguria]) [11]. Coronary artery disease was defined when coronary artery stenosis was greater than 70%. We defined early and late revascularization groups as patients who received percutaneous coronary intervention (PCI) or coronary artery bypass graft (CABG) either within or after 24 hours of the diagnosis of AMI, respectively.

### Data collection

Baseline clinical characteristics, including age, gender, chronic medical illnesses, infection site, and the duration between the diagnosis of AMI, and coronary revascularization, were collected. Sequential organ failure assessment (SOFA) and Glasgow Coma Scale (GCS) were calculated to evaluate disease severity. Time of diagnosis of AMI was defined as the first detection of elevated cardiac biomarkers. Cardiac biomarkers were measured in the central core laboratory when there were signs and symptoms of angina identified by the attending physicians. A consulting cardiologist interpreted electrocardiography. Troponin I was measured with Electrochemiluminescence immunoassay, and the 99^th^ percentile of upper reference limit value for this assay is 0.16 ng/ml. A cardiologist was consulted when the acute coronary syndrome was suspected by attending physicians. However, the timing of coronary revascularization was determined by the patient’s consulting cardiologist according to the patient’s conditions (i.e., age, symptoms, cardiovascular disease’s risk factors, hemodynamic status, electrocardiogram, laboratory data, echocardiographic parameters) and physicians’ preference. We compared the 30-day and one-year all-cause mortality between patients with early and delayed coronary revascularization.

### Statistical analysis

Categorial data was demonstrated as a number and percentages and compared by using the Chi-square test. Continuous data were shown as mean ± standard deviation and evaluated by the independent student T-test. We compared survival between early and delayed revascularization by Kaplan-Meier survival curves and the log-rank test. We used the cox proportional hazards regression model to calculate the hazard ratio (HR) and 95% confidence interval (95% CI). P values of less than 0.05 were considered to be statistically significant. All analysis was performed with SPSS statistical software (version 19.0; IBM Corporation, Armonk, New York, USA).

## Results

A total of 239 patients with non-ST elevation myocardial infarction after acute infection were evaluated. Baseline clinical characteristics of patients with early and delayed revascularization are displayed in Table 1. There were no significant differences in age; gender; co-morbidities such as diabetes mellitus, hypertension, hyperlipidemia, and infection site between the two groups. Patients in the early revascularization group were more likely to have higher initial cardiac biomarkers at the time of diagnosis, lower level of GCS scores, higher SOFA score, and reduced left ventricular ejection fraction. There was no difference in mechanical ventilation usage, renal replacement therapy, and hospitalization duration between the two groups.

**Table 1:** Baseline clinical characteristics of patients receiving early and delayed revascularization.

	Patients on early revascularization (n = 93)	Patients on delayed revascularization (n = 146)	P value
Age, years	76 ± 12	76 ± 12	0.737
Gender, male	73 (79)	111 (76)	0.753
Hypertension	66 (71)	111 (76)	0.449
DM	40 (43)	80 (55)	0.085
Hyperlipidemia	14 (15)	23 (16)	0.519
Prior stroke	14 (15)	15 (10)	0.312
ESRD	10 (11)	23 (16)	0.338
Prior CAD	21 (23)	36 (25)	0.757
Site of infection			0.259
Lung	44 (62)	71 (75)	
Genitourinary	8 (11)	6 (6)	
Gastrointestinal	9 (13)	6 (6)	
Others	10 (14)	12 (13)	
MAP, mmHg	80 ± 16	79 ± 17	0.625
WBC, /ul	12509 ± 4935	12079 ± 6043	0.566
Segment, %	80 ± 11	78 ± 12	0.182
Serum Cr, mg/dl	2.57 ± 2.15	2.87 ± 2.58	0.341
CRP	6.5 ± 7.1	6.1 ± 6.7	0.635
Initial CK	576 ± 689	330 ± 372	0.0001
Initial CK-MB	55 ± 57	29 ± 27	0.0001
Initial troponin I	19 ± 39	7 ± 11	0.001
LDL, U/l	84 ± 31	92 ± 36	0.216
Platelet, /ul	201624 ± 109861	206171 ± 89517	0.727
GCS	10 ± 4	11 ± 4	0.042
SOFA score	14 ± 8	12 ± 7	0.047
LVEF, %	29 ± 33	27 ± 26	0.398
On admission MV	61 (66)	84 (58)	0.225
On admission RRT	17 (19)	38 (27)	0.208
Hospital days	29 ± 33	27 ± 26	0.663

DM, diabetes mellitus; ESRD, end stage renal disease; MAP, mean arterial pressure; WBC, white blood cell; Cr, creatinine; CRP, C reactive protein; CK, creatine kinase; CK-MB, creatine kinase-MB type; LDL, low density lipoprotein; GCS, Glasgow coma scale; SOFA, sequential organ failure assessment; LVEF, left ventricular ejection fraction; MV, mechanical ventilation; RRT, renal replacement therapy.

Angiography disclosed no significant difference in the severity of coronary artery disease between the two groups (Table 2). There was a statistically insignificant increase in the prevalence of left main coronary artery disease in the early revascularization group. There was no difference in revascularization techniques (Percutaneous coronary intervention (PCI) or coronary artery bypass graft) between the two groups. (89% vs. 90%, P = 1.000 and 9% vs. 7%, P = 0.624 respectively).

**Table 2:** Angiographic characteristics between patients with early and delayed revascularization.

	Patient on early revascularization (n = 93)	Patient on delayed revascularization (n = 146)	P value
Disease severity			0.561
SVD	4 (4)	11 (8)	
DVD	19 (20)	26 (18)	
TVD	70 (75)	109 (75)	
LM	29 (31)	36 (25)	0.298
RCA	82 (88)	130 (89)	0.837
LAD	90 (97)	139 (95)	0.744
LCX	80 (86)	121 (83)	0.589
CTO	32 (34)	51 (35)	1.000
Revascularization modality			
PCI	83 (89)	131 (90)	1.000
CABG	8 (9)	10 (7)	0.624

SVD, single vessel disease; DVD, double vessel disease; TVD, triple vessel disease; LM, left main; RCA, right coronary artery; LAD, left anterior descending artery; LCX, left circumflex artery; CTO, chronic total occlusion; PCI: Percutaneous coronary intervention; CABG, coronary artery bypass graft.

**Fig 2:**
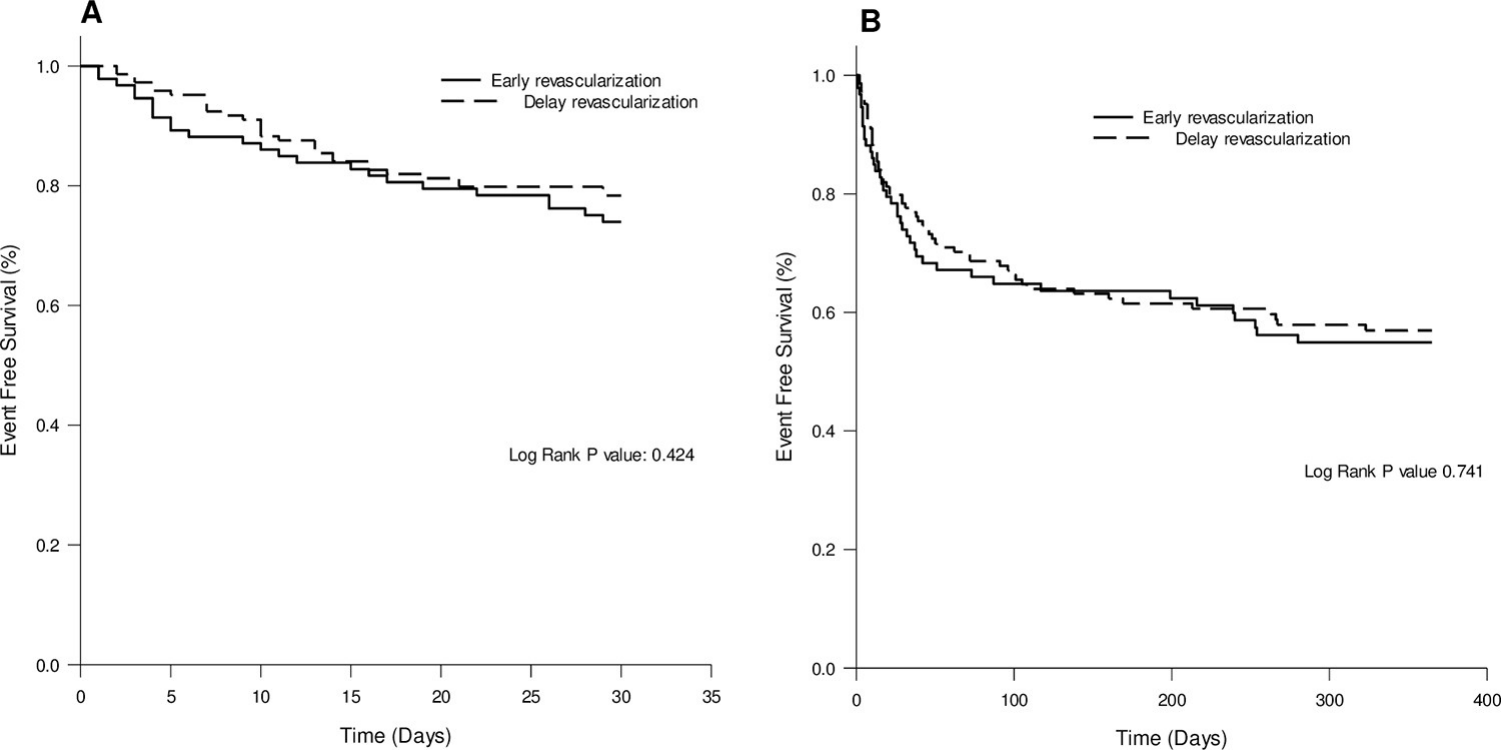
Kaplan-Meier survival curve of all-cause mortality of patients with post infectious ACS according to timing of revascularization. (A) 30-day all-cause mortality (B) one-year all-cause mortality.

There was no statistically difference in 30-day all-cause mortality (26% vs 22%, P = 0.532), one-year all-cause mortality (43% vs 40%, P = 0.788), one-year cardiovascular mortality (27% vs 25%, P = 0.761) and one-year non-fatal MI (2% vs 1%, P = 0.644) between early and delayed revascularization groups. (Table 3) A Kaplan-Meier survival curve of short- and long-term all-cause mortality stratified by timing of revascularization is shown in Fig 2.

**Table 3:** Outcomes of patients with early and delayed revascularization.

	Patients on early revascularization (n = 93)	Patients on delayed revascularization (n = 146)	P value
30-days all-cause mortality	24 (26)	32 (22)	0.532
One-year all-cause mortality	40 (43)	59 (40)	0.788
One-year CV mortality	25 (27)	36 (25)	0.761
One-year non-fatal MI	2 (2)	2 (1)	0.644

CV, cardiovascular; MI, myocardial infarct.

In univariate analysis, age, shock at the time of diagnosis of AMI, initial troponin I level, mechanical ventilation use, GCS and SOFA scores at the time of AMI diagnosis, coronary artery disease severity were significant predictors of one-year all-cause mortality. In multivariate Cox regression analysis, age, mechanical ventilator use, GCS, and SOFA score remained statistically significant indicators for one-year all-cause mortality (Table 4).

**Table 4:** Univariate and multivariate analysis of risk factors of one-year all-cause mortality.

	Univariate analysis		Multivariate analysis	
	HR (95% CI)	P value	HR (95% CI)	P value
Time of revascularization (early vs delay)	0.935 (0.626–1.397)	0.742		
Age	1.033 (1.012–1.053)	0.001	1.030 (1.009–1.051)	0.004
Gender	1.151 (0.731–1.811)	0.545		
Hemodynamic unstable	2.672 (1.771–4.032)	0.0001	1.489 (0.921–2.407)	0.104
Hypertension	0.996 (0.637–1.560)	0.987		
DM	1.282 (0.861–1.908)	0.221		
Hyperlipidemia	0.857 (0.487–1.509)	0.594		
Prior stroke	1.312 (0.745–2.311)	0.346		
ESRD	1.180 (0.691–2.015)	0.545		
Initial troponin I	1.007 (1.002–1.012)	0.011	1.004 (0.998–1.010)	0.226
CRP	0.994 (0.964–1.025)	0.709		
LVEF	0.987 (0.964–1.009)	0.243		
On admission MV	1.611 (1.057–2.457)	0.027	0.501 (0.277–0.909)	0.023
On admission RRT	1.320 (0.836–2.085)	0.233		
GCS	0.891 (0.850–0.934)	0.0001	0.902 (0.840–0.968)	0.004
SOFA score	1.073 (1.045–1.102)	0.0001	1.059 (1.020–1.098)	0.002
Coronary artery severity	1.566 (1.043–2.352)	0.031	1.273 (0.848–1.913)	0.245
Presence of CTO lesion	1.410 (0.943–2.107)	0.094		

DM, diabetes mellitus; PCI, percutaneous coronary intervention; ESRD, end stage renal disease; LVEF, left ventricular ejection fraction; MV, mechanical ventilation; RRT, renal replacement therapy; GCS, Glasgow coma scale; SOFA, sequential organ failure assessment.

## Discussion

In this retrospective, observational cohort study, patients with higher cardiac biomarkers at symptom onset and decreased conscious level received invasive management within 24 hours. There was no statistically significant difference in 30-day and one-year all-cause mortality between early and delayed coronary revascularization strategies.

The myocardial injury commonly complicates severe sepsis or septic shock, ranging from 12% to 85%. Systemic infection may lead to endothelial dysfunction, platelet activation, and destabilization of atherosclerotic plaques [6]. Besides, sepsis promoted oxygen demand and leads to supply demand mismatch [7]. Elevated troponin levels are associated with increased mortality and length of hospital stay [12]. Long-term prognosis is also worse when infection accompanies AMI [8]. The differentiation of type 1 myocardial infarction (obstructive coronary artery disease) from type 2 myocardial infarction (demand ischemia) in patients with an initial presentation of sepsis can be challenging. In this situation, if patients had peripheral vascular disease or at least two cardiovascular risk factors, odd ratios to have obstructive coronary artery disease were 5.7 (95% CI, 1.1–30.4, P = 0.042) and 6.7 (95% CI, 1.9–23.8; P = 0.003) [13]. The primary treatment options for type 1 myocardial infarction are myocardial revascularization with either PCI or CABG combined with thrombolytic therapy [10]. However, there are no specific management guidelines for post-infectious AMI. Invasive coronary revascularization with PCI or CABG was reported to have a survival benefit in these patients (HR 0.62, 95% CI 0.60–0.65), in an observational study [8].

In patients with ST-elevation myocardial infarction, the 2018 European Society of Cardiology guidelines for myocardial revascularization recommended door to wire crossing of <90 minutes in non-PCI center and <60 minutes in high-volume PCI centers. For non-ST elevation myocardial infarction, coronary revascularization was suggested as immediate (<2 hours), early (<24 hours), and delay (> 24 hours) invasive strategies depending upon risk factors such as cardiogenic shock, ongoing chest pain, life-threatening arrhythmia, mechanical complications from myocardial infarction, acute heart failure, dynamic ST changes, Global Registry of Acute Coronary Events score, diabetes mellitus, chronic kidney disease [10]. However, the optimal timing of coronary angiography and intervention in septic patients combined with myocardial infarction remains elusive.

Revascularization decreased death and recurrent myocardial infarction at 6 months compared to medical therapy alone in patients with non-ST-segment elevation myocardial infarction [14]. The timing of coronary angiography was different among centers, ranging from 19 hours to 96 hours. However, early and delayed intervention strategies were not found to yield different results in the composite outcome of death, myocardial infarction, or refractory ischemia at 6 months.

Winter RJ et al. found that routine early invasive revascularization didn’t lower mortality in patients with non-ST elevation myocardial infarction in a randomized trial. Moreover, routine early revascularization may carry a further risk of myocardial infarction [15]. Moreover, in another randomized control trial, a composite endpoint of death and non-fatal myocardial infarction was found to be higher at one month in the early routine revascularization group. However, there was no significant difference at one year compared to conservative revascularization [16]. Mehta SR et al. also reported that delayed invasive intervention beyond 72 hours didn’t significantly affect outcomes of mortality, myocardial infarction, or stroke in the long term [17].

Of importance, we observed that delayed coronary revascularization 24 hours after symptom onset might not worsen short and long-term outcomes. These patients may require the initial stabilization of sepsis before invasive coronary management [18]. Surviving sepsis campaign suggested sepsis as a medical emergency similar to AMI and stroke, requiring immediate resuscitation and antibiotic treatment [19]. Timely administration of the first dose of antibiotic, identification of the primary infection site, and adequate fluid resuscitation are crucial components of the management of sepsis. A delay of over one hour in administering the first dose of adequate antibiotics therapy is an independent predictor of 28-day mortality [20]. Active infection and inflammation may play roles in promoting coronary stent thrombosis [21]. Consequently, most physicians deferred invasive revascularization in acutely infected patients. Coronary angiography is typically deferred up to three days after symptom onset in patients with post-infectious AMI [13].

Contrast-induced nephropathy after percutaneous coronary intervention carries a poor long-term prognosis and is always a great concern in arrangement of coronary intervention in septic patients [22]. The incidence of acute kidney injury is highest in the setting of AMI and low left ventricular ejection fraction [23]. High levels of inflammatory biomarkers, such as high-sensitivity C-reactive protein or procalcitonin, which are frequently elevated during systemic infection, are also associated with contrast-induced nephropathy [24].

Our study had several limitations. First, the duration of myocardial ischemia may not be accurately determined, especially in patients receiving mechanical ventilation or with altered levels of consciousness from severe sepsis, as our case definition of post-infectious AMI is based on the first abnormal laboratory time point. Although there was no difference in usage of mechanical ventilation between the two groups, there may be a potential risk of delayed diagnosis. Secondly, when to revascularize was determined by a consulting cardiologist and attending physicians. However, in real-world practice, there are no explicit guidelines to follow in post-infectious AMI. Moreover, which kind of treatment (i.e., either to receive PCI or CABG) to be received was decided jointly by patient, cardiologist and cardiovascular surgeon. It may lead to possible selection bias. Nevertheless, there was no statistically significant difference of treatment strategies between two groups in our study. Thirdly, patients with higher first serum troponin I level, unstable hemodynamic status, lower left ventricular ejection fraction may have received early revascularization while their prognosis was generally poorer. There may be selection bias. Fourthly, most of our patients didn’t receive intracoronary imaging during intervention to identify intraluminal thrombus from plaque rupture, ulceration, erosion or dissection so it is difficult to differentiate type 1 and type 2 AMI. Lastly, it is a single, centered retrospective study.

## Conclusions

In patients with post infectious AMI, coronary revascularization may be arranged according to individual’s risk category as those without sepsis.
